# Force Systems Produced by Different Cantilever Configurations during Deactivation

**DOI:** 10.3390/ma15144815

**Published:** 2022-07-10

**Authors:** Malgorzata Bilinska, Isabel Meret Golliez, Michel Dalstra

**Affiliations:** 1 Section of Orthodontics, Department of Dentistry and Oral Health, Aarhus University, Vennelyst Boulevard 9, 8000 Aarhus, Denmark; michel.dalstra@dent.au.dk; 2 Department of Pediatric Oral Health and Orthodontics, University Center for Dental Medicine UZB, University of Basel, Mattenstrasse 40, 4058 Basel, Switzerland; isabel.golliez@gmx.ch

**Keywords:** orthodontics, biomechanics, cantilevers

## Abstract

Intrusion with a three-piece arch is routinely achieved during orthodontic treatment. This study aimed to experimentally determine how the cantilever design influences the generated force system. Both straight and arch-formed cantilever designs: tip-back (TB), flat curve (FC) deep curve (DC), and 3 mm and 6 mm high utility arch (UA3; UA6) were activated for 5 mm and 10 mm. Force systems were determined by a hexapod. Typodonts simulating a three piece-intrusion arch were scanned using an intraoral scanner (3Shape, TRIOS, Denmark) before (T0) and after (T1) the experiment and superimposed with Mimics software (Materialise, Leuven, Belgium). Data were analyzed. All straight designs displayed an extrusive force in the vertical plane, and all arch-formed an intrusive force. DC and TB showed a retrusive force in the sagittal plane and UA6 a protrusive. For the medial/lateral forces, DC and TB displayed a medial, and UA6 a lateral force. Configurations can be distinctively ranked from DC, FC, TB to UA3, and UA6 according to the increasing protrusive nature of the generated sagittal forces. A DC or TB configuration should be used for intrusion and retraction, while for an intrusion and a protrusion, a UA6 configuration. All straight configurations showed a higher force level than the arch-formed configurations.

## 1. Introduction

**Figure 1:**
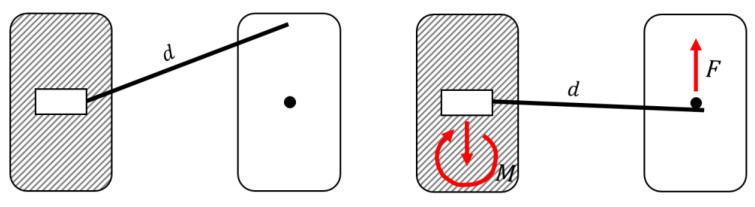
Force system of a cantilever. Left: Cantilever in a neutral position with a specific length (*d*). Right: Activated cantilever generating a single force (*F*) on one side and a force and a moment (*M*) on the other side.

Cantilevers are an efficient choice from a biomechanical point of view because all forces can be measured and thus controlled. Produced force systems are statically determined; the generated force is a one-point contact, while in the reactive unit, an opposite direction of force and moment occur [1] (Figure 1). Besides vertical activation, cantilevers can also be activated to produce a palatal as well as a buccal force [2,3,4]. They may serve as a simple system to solve multiple orthodontic problems [2,5], including frontal segment intrusion in deep bite correction [5,6] uprighting of tipped molars [7,8], intrusion of a single tooth [9,10], treatment of impacted teeth [11,12,13,14,15], correction of dental transposition [16,17,18,19], space closure [20], correction of occlusal plane cant [21] and dental asymmetries [22]. The commonly used material to manufacture cantilevers is titanium molybdenum alloy (TMA). TMA wires were introduced to orthodontics in 1979 by Goldberg and Burstone [23]. TMA alloys have several advantages, including excellent formability, biocompatibility, and an elastic modulus below stainless steel. However, their high surface roughness and susceptibility to fracture during bending narrow the clinical indications [24].

In the literature, studies considering force delivered by cantilevers are limited, as well as the asymmetric and symmetric cantilevers activation. Melsen et al. evaluated the force system produced by stainless steel and beta-titanium cantilevers with an eccentrically placed helix [25]. A study by Dalstra and Melsen (1990) evaluated force systems developed by six different cantilever configurations used as part of a three-piece intrusion mechanics. The results were investigated using the Finite Element Method. Bilinska and Dalstra (2022) evaluated in vitro the resultant horizontal and vertical forces, delivered with asymmetric and symmetric loading of the frontal segment with regard to possible use for midline correction [26]. A study by Jacob et al. appraised the force decay and design shape changes caused by stress relaxation in two different orthodontic cantilever configurations [27].

Specific cantilever shapes and forms influence the delivered force system. A study by Dalstra and Melsen indicated that depending on the configuration, the resultant force may differ. Initial protrusive forces were found in utility-arch-formed cantilevers, while curved cantilevers directly generated a retrusive force [1]. Depending on the clinical examination and treatment plan, in the correction of deep bite, a segmented approach to intrude and retract the incisors is clinically advantageous. It allows simultaneous control of tooth movement in both vertical and anteroposterior planes [28]. When the incisors are retroclined, proclination and intrusion would be desired. Yet, since in a clinical setting the cantilevers are arch-formed, it is essential to know how the force systems perform in a three-dimensional setting. The resultant force of different cantilever configurations would help to choose the “best-fit” cantilever configuration for specific clinical indications.

The present study aims to determine the influence of different types of cantilever design on the components of the deactivation force in the sagittal, horizontal and vertical axes in an experimental setting. The study was designed to assess the difference with 3D arch-formed cantilevers, which was not addressed in the study of Dalstra and Melsen (1999) [1].

## 2. Materials and Methods

**Figure 2:**
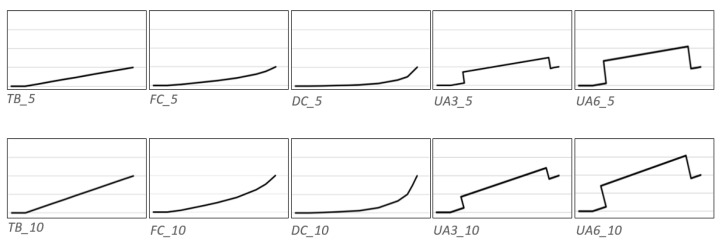
Template of the cantilever configurations in the sagittal view.

Twenty different cantilevers designs were included in the study, originating from Dalstra and Melsen’s (1999) study [1]. All cantilevers were made from beta-titanium (TMA) 0.017″ × 0.025″ (American Orthodontics, REF 73-L000-1725, Sheboygan, WI, USA). Tip back (TB), deep curve (DC), flat curve (FC), 3 mm high utility arch (UA3), and 6 mm high utility arch (UA6) designs were applied. In the vertical dimension, the activation range was 5 mm and 10 mm (Figure 2).

**Figure 3:**
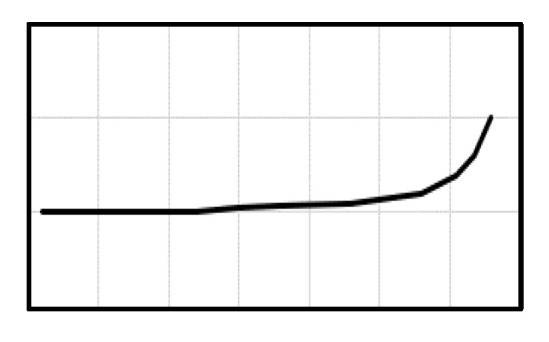
Arch-formed configurations in the occlusal view.

Furthermore, in the occlusal plane, the cantilevers were either straight or arch-formed (Figure 3). In clinical practice, a straight shape is applied less frequently compared to the arch formed, as it can impinge with the gingiva upon activation. Therefore, 20 different cantilever designs were analyzed (Table 1). For all 20 designs, five copies were produced. In total 100 cantilever samples were fabricated.

**Figure 4:**
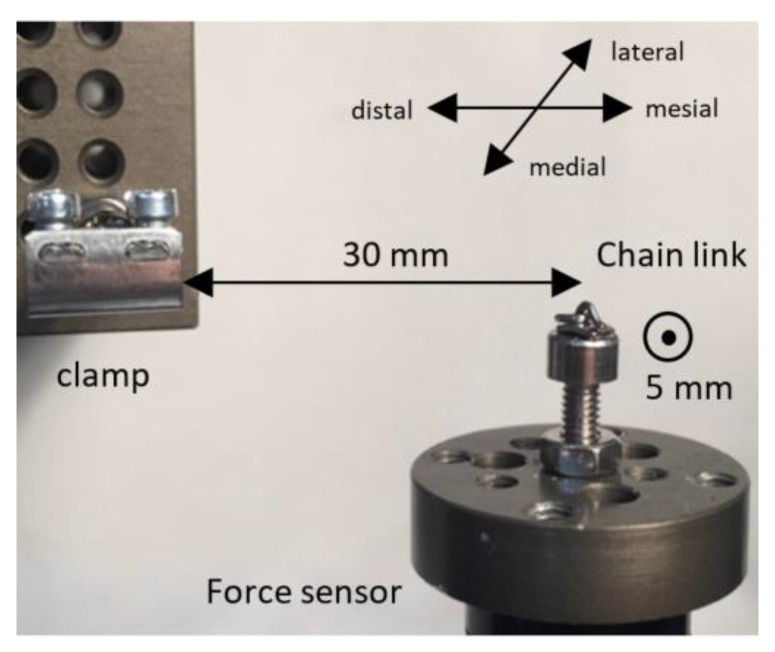
The experimental set-up with the relevant directions and dimensions.

Measurements were performed on a hexapod PAROS II (Paros II Hexapod, Physics Instruments, Auburn, MA, USA). A force sensor (FDT Nano 17 force sensor, Physics Instruments, Auburn, MA, USA) with a single chain link was installed to engage the mesial hook of the cantilever. The single chain link was chosen to emulate a clinically used metal ligature to connect the buccal segment to the anterior segment. At the static part of the hexapod, a clamp to place the distal end of the cantilever was attached. The chain link was then placed on the same horizontal level as the clamp. In the occlusal view, the chain link was placed at 30 mm mesial and 5 mm medial according to the predefined arch form (Figure 4).

**Figure 5:**
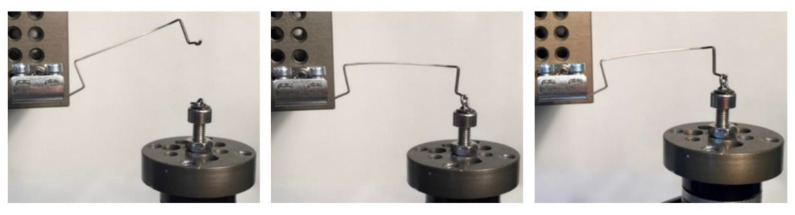
The three relevant positions of the cantilever during the measurements showed by the example of a 6 mm high utility arch. Left: neutral. Middle: activated. Right: 3 mm deactivated.

For the measurements, the distal end of the cantilever was fixed to the clamp first, subsequently, the cantilever was activated by engaging the mesial hook into the chain link. The movable platform with the force sensor was then moved three millimeters apically with a predefined inclination (Figure 5).

**Figure 6:**
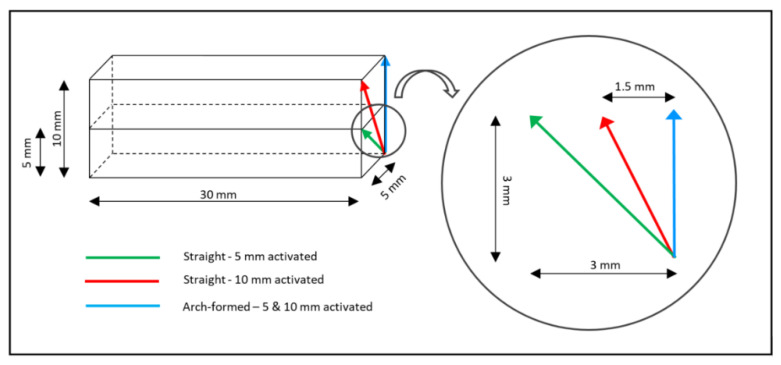
Scheme showing the deactivation trajectory used for the different shape/activation combinations. Note that the common starting point of the three trajectories represents the point of maximal activation.

The trajectory of the movement of the force sensor to simulate the deactivation depended on the tested configuration. For the arch-formed configurations, the sensor was moved straight vertically upwards. For the straight and 5 mm activated configurations, the movement was 3 mm horizontal and 3 mm vertical, while for the straight and 10 mm activated configurations, the movement was 1.5 mm horizontal and 3 mm vertical (Figure 6). The velocity of the movement was 10 mm/s for all configurations.

**Figure 7:**
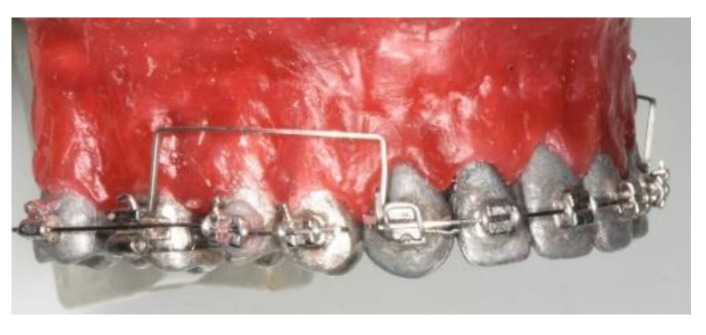
Cantilevers (UA6) applied on the typodont.

Three arch-formed cantilever designs: deep curve, tip back, and utility arch shape cantilevers were experimentally tested on a typodont (artificial teeth embedded in wax) simulating bilateral, symmetric intrusion of the anterior segment in the upper arch. The chosen designs are most commonly used in the clinical practice and for this reason, were included in the experimental part of the study. The arched form was chosen to avoid interference with the brackets. The design is analogous to Burstone’s three-piece intrusion arch, with a 0.018″ × 0.025″ stainless steel segmented wire for anterior and posterior segments and a 0.08″ SS transpalatal arch (TPA) to provide anchorage [6,26]. The cantilevers were placed in the auxiliary tubes on molars bands and ligated on the anterior segment, distally to the canines (Figure 7).

The cantilevers were activated for intrusion for 5 and 10 mm, measured as a distance from the activated cantilever to the ligation point [26]. Typodonts with activated cantilevers were bathed for 5 min in water with a temperature of 60 degrees Celsius and cooled down to room temperature. The water temperature, controlled with an electronic thermometer, remained stable throughout the experiment. Scans of all typodonts were performed (3Shape, TRIOS, A/S, Copenhagen, Denmark); before the cantilevers were placed (T0) and after the experiment was completed (T1). Superimposition of the T0 and T1 scans was performed using Mimics software (Materialise, Leuven, Belgium). Tests were repeated two times to assess the repeatability. As six teeth were present in the anterior segment, all teeth was judged separately (no movement/displacement in transversal plane/sagittal plane). Data from the experimental part were analyzed qualitatively.

### Data Analysis

**Figure 8:**
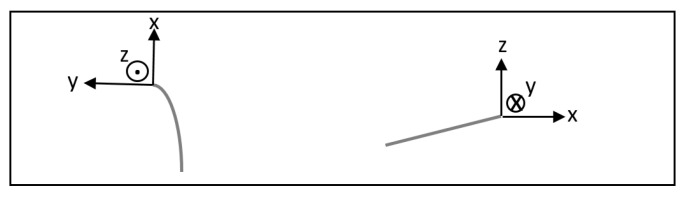
The coordinate system at the anterior end of the cantilever with the directions of the positive x-, y-, and z-axes. (**Left**) occlusal view. (**Right**) sagittal view.

The analysis of the data was performed in Excel 2016 (Microsoft, Redmond, WA, USA). To analyze the relation between force and deactivation, a second-order polynomial regression was fitted to the data. For six defined points of deactivation (0 mm, 0.5 mm, 1.0 mm, 1.5 mm, 2.0 mm, 2.5 mm, and 3.0 mm) the force was calculated and plotted in graphs. For each cantilever design, the test was repeated for five different copies; the average force/deactivation graph was determined as well as the 95% confidence interval (CI) around it. A significant difference between the two cantilever designs was assumed when the CIs of these designs would not overlap each other during the first 3 mm of deactivation. The coordinate system used to evaluate the direction of the forces is shown in Figure 8.

## 3. Results

### 3.1. Sagittal Forces

#### 3.1.1. Arch-Formed and 5 mm Activation

**Figure 9:**
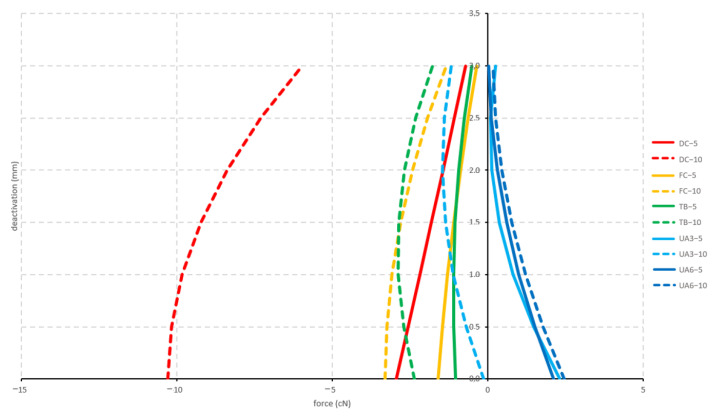
Sagittal forces generated by the arch-formed configurations (see Figure 2) during deactivation (protrusive; positive values; retrusive: negative values). Tested cantilever configurations origin from Figure 2.

Both curved configurations (deep curve and flat curve) and the tip-back configuration displayed a retraction, while both utility configurations (3 mm high and 6 mm high) displayed a protrusion. There is no significant difference between the forces generated by these five configurations, as the CIs of the various configurations overlapped each other. The produced forces by the arch-formed and 5 mm activated configurations ranged between −2.9 cN (deep curve) and 2.8 cN (6 mm high utility arch) (Figure 9).

#### 3.1.2. Arch-Formed and 10 mm Activation

All configurations displayed a retraction except for the 6 mm high utility configuration, which displayed a protrusion. There was no significant difference between the forces generated by these five configurations. The maximum forces produced by the arch-formed and 10 mm activated configurations were −9.1 cN (deep curve) and 3.0 cN (6 mm high utility) (Figure 9).

#### 3.1.3. Straight and 5 mm Activation

**Figure 10:**
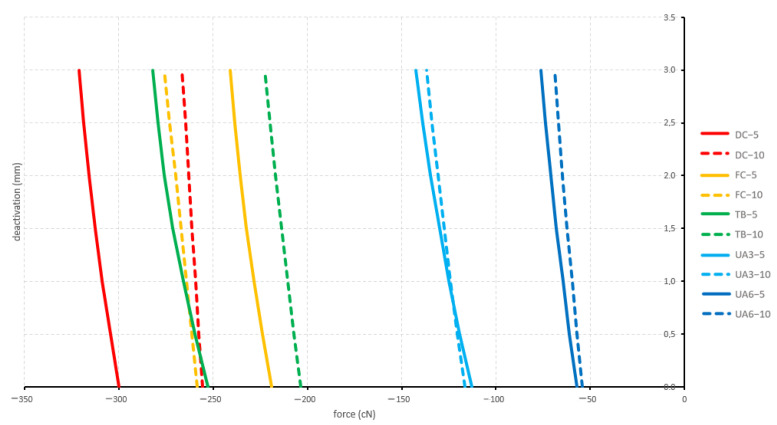
Sagittal forces generated by the straight configurations during deactivation (protrusion positive; retraction negative). Tested cantilever configurations origin from Figure 2.

All straight and 5 mm activated configurations displayed a retraction. Both curved configurations and the tip-back configuration showed significantly different forces during deactivation compared to both utility arches. The produced forces by the straight configurations ranged between −321.1 cN (deep curve) and −57.2 cN (6 mm high utility arch) (Figure 10).

#### 3.1.4. Straight and 10 mm Activation

All straight and 10 mm activated configurations displayed a retraction. Like above for the 5 mm activation, both curved configurations and the tip-back configuration showed significantly different forces during deactivation compared to both utility arches. Additionally, the flat curve showed significantly different forces during deactivation compared to the tip-back configuration. The produced forces by the straight configurations ranged between −276.0 cN (flat curve) and −54.2 cN (6 mm high utility arch) (Figure 10).

### 3.2. Vertical Forces

#### 3.2.1. Arch Formed

**Figure 11:**
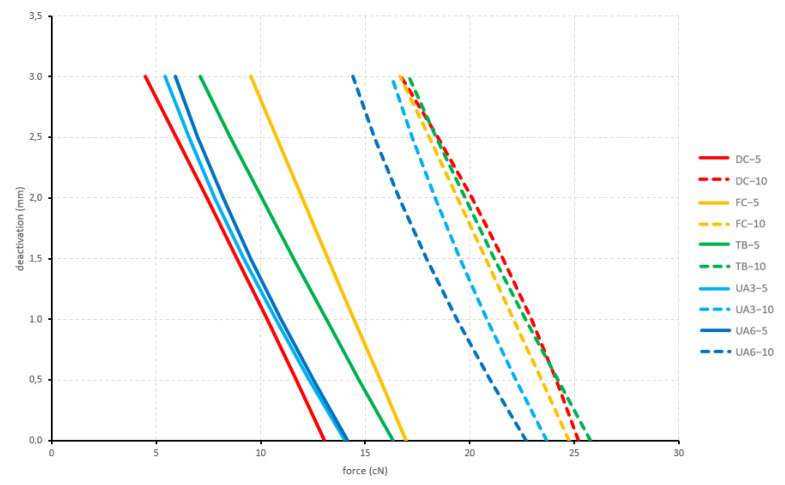
Vertical forces generated by the arch-formed configurations during deactivation. Tested cantilever configurations origin from Figure 2.

All configurations displayed an intrusive force with no significant differences between the produced forces. The force was constantly decreasing during deactivation. The 10 mm activated configurations displayed a mean force of 20.3 cN and the 5 mm activated configurations displayed an initial mean force of 10.6 cN (Figure 11).

#### 3.2.2. Straight

**Figure 12:**
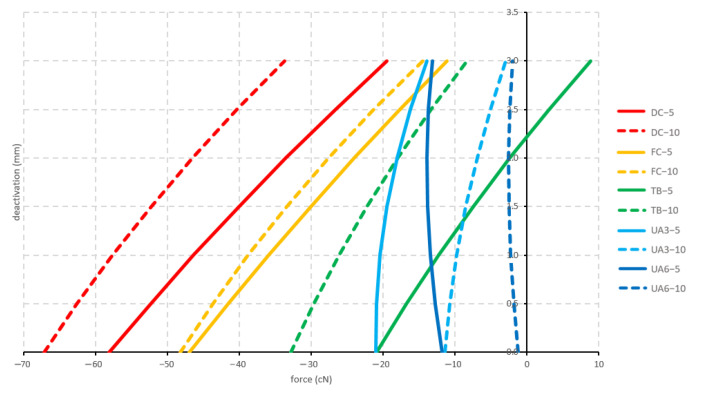
Vertical forces generated by the straight configurations during deactivation. Tested cantilever configurations origin from Figure 2.

Initially, all straight configurations displayed an extrusive force. The only significant different forces in this group were between the 10 mm activated deep-curved configuration and the 10 mm activated 6 mm high utility arch configuration. After 2 mm deactivation, the tip-back 5 mm activated configuration changed from an extrusive force into an intrusive force. During deactivation, the forces constantly decreased except for both 6 mm high utility configurations (Figure 12).

### 3.3. Medial/Lateral Forces

#### 3.3.1. Arch-Formed and 5 mm Activation

**Figure 13:**
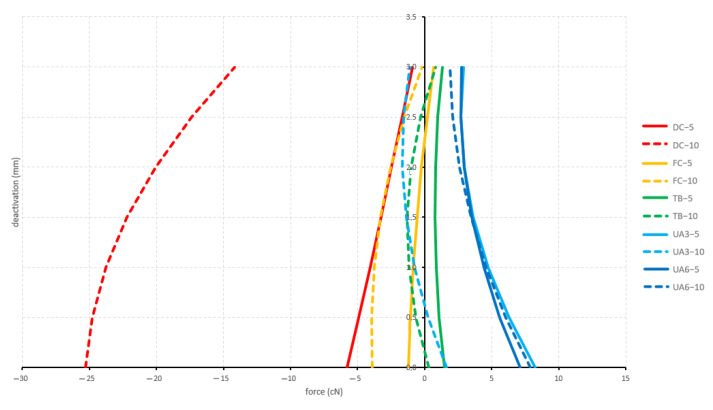
Medial/lateral forces are generated by the arch-formed configurations during deactivation (medial positive; lateral negative). Tested cantilever configurations origin from Figure 2.

Both utility configurations displayed a medial force, while the deep-curved configuration displayed a lateral force. The tip-back configuration and the flat-curved configuration started with a lateral force and changed into a medial force after 2 and 2.5 mm of deactivation, respectively. There was no statistically significant difference between the forces generated by the arch-formed and 5 mm activated configurations. The forces produced by the arch-formed and 5 mm activated configurations ranged between −5.8 cN (deep curve) and 8.6 cN (6 mm high utility arch) (Figure 13).

#### 3.3.2. Arch-Formed and 10 mm Activation

Both curved configurations displayed a lateral force, while the 6 mm high utility configuration displayed a medial force. The 3 mm utility configuration displayed at first a medial force and then changed into a lateral force. The tip-back configuration displayed at first a medial force then changed into a lateral force, and finally changed into a medial force again. There was no statistical difference between the forces generated by the arch-formed and 10 mm activated configurations. The produced forces by the arch-formed and 10 mm activated configurations ranged between −21.2 cN (deep curve) and 9.8 cN (6 mm high utility arch) (Figure 13).

#### 3.3.3. Straight and 5 mm Activation

**Figure 14:**
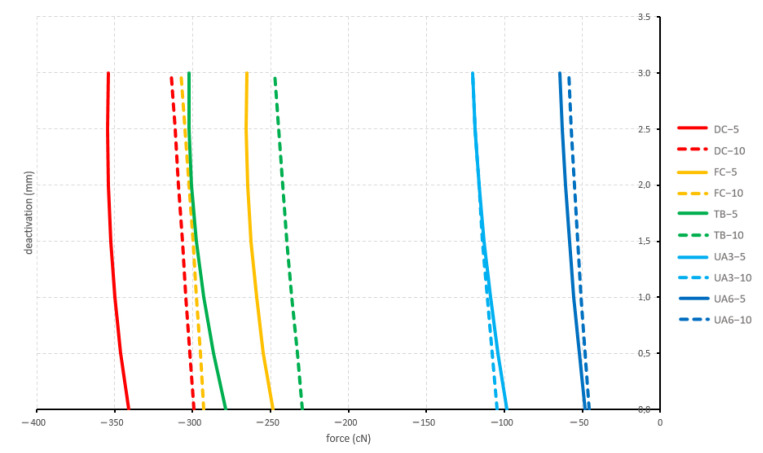
Medial/lateral forces generated by the straight configurations during deactivation (medial positive; lateral negative). Tested cantilever configurations origin from Figure 2.

All straight configurations displayed a lateral force. Both curved configurations and the tip-back configuration showed significantly different forces during deactivation compared to both utility arches. The produced forces by the straight and 5 mm activated configurations ranged between −354.6 cN (deep curve) and −64.7 cN (6 mm high utility arch (Figure 14).

#### 3.3.4. Straight and 10 mm Activation

**Figure 15:**
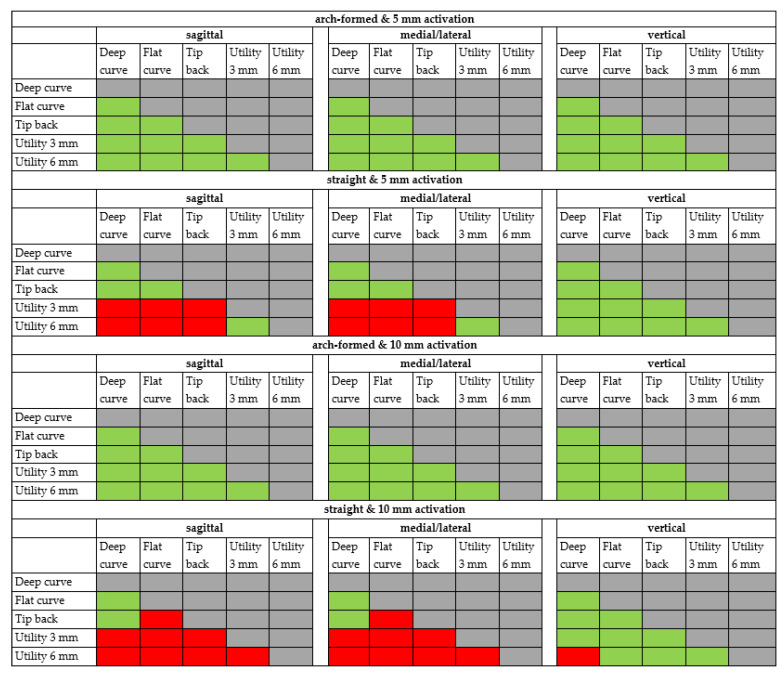
Comparison of the cantilever configurations by the level of significant difference. red = significant difference, green = no significant difference.

Additionally, here, all straight configurations displayed a lateral force. Both curved configurations and the tip-back configuration showed significantly different forces during deactivation compared to both utility arches. Additionally, the flat curve showed significantly different forces during deactivation compared to the tip-back configuration. The produced forces by the straight and 5 mm activated configuration lay between −313.3 cN (deep curve) and −58.8 cN (6 mm high utility arch) (Figure 14). All sagittal forces of any configuration showed a strong correlation to its medial/lateral forces. The correlation factors ranged between R = 0.8795 and R = 0.9999 (Figure 15). According to the direction of the force components described above, the forces can be classified as being protrusive, retrusive, medial, lateral, or intrusive (Table 2). Resultant forces in all three planes of space are summarized in Table 3 and Table 4.

Summing up the abovementioned statistically significant differences between the force components generated by the five different configurations yields the following overview (Figure 15).

### 3.4. Experimental Part: Typodonts

**Figure 16:**
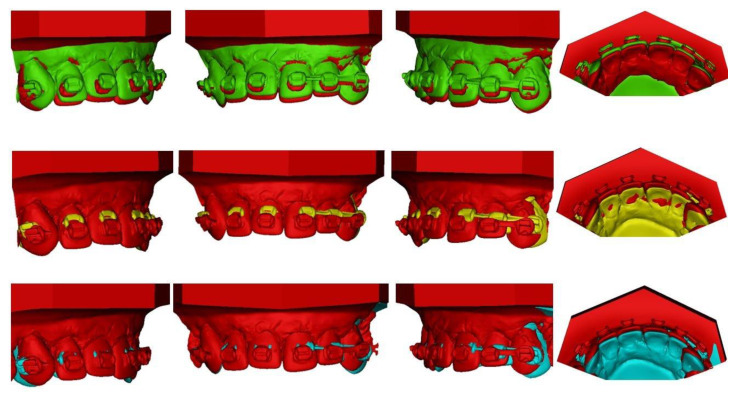
Superimpositions of typodonts in 10 mm cantilever activation: UA6 (red: T0, green: T1): intrusion and protrusion observed, TB (red: T0, yellow: T1): intrusion and retrusion observed DC (red: T0, blue: T1): intrusion and retrusion; retrusion more pronounced for DC than for TB.

The results from the experimental part were consistent with the measurements. The bilateral placement of TB and DC resulted in both retraction and intrusion, and UA6 in protraction and intrusion. Both 5 and 10 mm activation resulted in the same result, with a different range of tooth movement. Activation for 5 mm produced much less movement compared to 10 mm: the intrusion was minor and almost clinically insignificant. Additionally,10 mm activation resulted in intrusion and retraction (TB, DC) and intrusion and protraction (UA6). The resultant retraction of the front segment was more pronounced for DC than for TB. With the application of a TB cantilever, the retraction of the frontal segment was diminished and the most pronounced effect was the intrusion, with a slight retrusive component. The results are consistent with force measurements. As the cantilevers were placed symmetrically, the same resultant movement was found on both sides of the anterior segment. The results are presented in Figure 16. In both 5 and 10 mm activation, the effect on the anchorage unit (molars) was negligible. The tests were performed twice: the models were superimposed and the tooth movement was evaluated. The repeatability was assessed as very good, because the results were the same for all teeth evaluated.

## 4. Discussion

The purpose of the study was to determine whether there is a difference in the force systems produced by various orthodontic cantilever designs during deactivation. The considered design variations in this experimental study were tip back, deep curve, flat curve, 3 mm high utility arch, and 6 mm high utility arch.

All cantilevers were activated for intrusion at the distal part. The study by Jacob et al. evaluated different preactivations designs to evaluate the force decay. Tested cantilevers were utility-shaped. They concluded that the design of the cantilever and proximity of the bends influenced the force decay pattern over time. Cantilevers activated at the distal part—similar to our study—presented a more stable design in the first weeks compared to the activation located at the bend located inferiorly at the utility shape [27].

In the current study, cantilevers were both arch-formed and straight. The straight form is rarely used in clinical practice, because it can impinge on the gingiva upon activation and cause interference with the brackets. However, it may be applied on the palatal side of an appliance and deliver traction to resolve dental impaction [11,14,29]. A study by Melsen et al. [25] analyzed the force direction delivered by stainless steel and beta-titanium cantilevers with an eccentrically placed helix. The results confirmed that the wire configuration delivers a predetermined combination of horizontal and vertical force. In the current study, the forces generated by the straight configurations were generally higher than the forces generated by the arch-formed configurations. This was expected since the arch-formed configurations were already bent into the arch form and only had to be activated in the vertical direction to be hooked into the chain link. The initial length of the wire of the straight and corresponding arch-formed cantilevers was the same to keep the comparison valid. However, it was not expected that the vertical forces for the straight cantilevers were extractive. This would imply that immediately upon release after full activation, the active unit would feel the tendency to be pulled even further down, rather than upwards to its undeformed state. The explanation can be found in the way how the straight cantilevers are deformed during activation. As the bending of the wire occurs in two planes (vertically either 5 or 10 mm and medially 5 mm), an additional torsional warping of the cantilever has to occur to reach the endpoint of activation. When the hook of the cantilever is fixed in this position (and deactivation is about to commence), the cantilever wants to untwist, thereby producing an extra force going against the expected intrusive force. Over the first 3 mm of deactivation, the extractive nature of these vertical forces quickly decreases though (Figure 12). In this warping behavior of the deformed wire, the cause of the non-proportional behavior of the 10 versus 5 mm activation for the straight cantilevers also has to be found: the dual nature of the simultaneous bending and twisting creates geometrical non-linearities, which no longer follow apparent and/or intuitive rules. Another difference between straight and arch-formed cantilevers is the relative size of the vertical force in comparison to the horizontal force components. For the arch-formed cantilevers, the vertical force is the main component, to which the sagittal and medial/lateral components are subject. For the straight cantilevers, it is the opposite. The “swing-door” effect of the straight cantilevers in the medial/lateral aspect upon deactivation almost overrides the vertical activation and results in large sagittal and medial/lateral forces.

Arch form cantilevers are commonly applied in the auxiliary tube of the molar and the corrected tooth/segment [3,4,6,17]. A long cantilever arm can deliver a relatively low load-deflection rate. Produced force system may be also used for root uprighting as the high moment occurs [30].

The deep curve produced the most retrusive and medial forces, while the 6 mm high utility arch produced the most protrusive and lateral forces. The tip-back produced less retrusion and intrusion. For the sagittal forces, this sequence was consistent with the study of Dalstra and Melsen (1999); the medial/lateral forces had not been considered by them, due to the 2D nature of their study [1]. The order of this ranking is determined by the way how the cantilever is being deformed during activation. The free end of an activated deep-curve cantilever moves posteriorly upon deactivation, while the free end of a utility-arch cantilever tends to move slightly anterior during the initial phase of deactivation. The magnitude of the forces, in general, was so low for the arch-formed cantilevers, that the 95% CI of the force curves of the configurations overlapped each other, so no significant differences between the configurations were found, even though the order of the ranking remained present. As to be expected, 5 mm activation produced smaller forces than 10 mm for the examined cantilevers. However, apart from the vertical forces, this effect was not proportional. Activation of 10 mm produced only a marginally larger protrusive force for the 6 mm high utility arch than 5 mm activation, while the same increase in activation had a much larger effect on the retrusive force of the deep curve. The result was confirmed in an experimental setting, where activation of 5 mm produced less effect compared to 10 mm. In the literature, recommended the range of forces to intrude the upper front segment varies between 60 and 150 g [6,22,31]. According to van Steenbergen, the force for maxillary incisor intrusion varies between 10 and 20 g per tooth. However, an intrusive force of 80 g did not increase the intrusion rate when compared with 40 g and no significant difference in buccal segments extrusion was observed between the two forces used [22]. According to Shroff, low load deflection springs will deliver optimal magnitudes of force for anterior segment intrusion; heavier forces will not increase the rate of intrusion. It aids to minimize root resorption and decrease side effects on the reactive unit [28]. Cantilevers and three-piece archwires are successful clinical applications of TMA wire as they provide an efficient working range for tooth movement. In the current study, all cantilevers were manufactured by beta-titanium wire from the same batch to avoid differences between the wires. However, a study by Gurgel et al. analyzed the force-deflection behavior of six beta-titanium wires in the bending test. Significant differences were observed, indicating the presence of different forces for the same amount of deflection. For future research, the evaluation of the delivered force by cantilever made of different beta-titanium alloys may improve the alloy choice for specific clinical indications [24].

### 4.1. Anchorage Unit Considerations

The static part of the hexapod represents the anchorage unit of a clinical situation. An intrusive force at the anterior results in a distally tipping moment at the distal end. Sagittal forces will generate moments mesially/distally rotating around the longitudinal axis of the anchorage unit. The last of the three orthogonal moment components is a rotation: the intrusive forces for the arch-formed cantilevers will generate buccally directed tipping moments. In the typodont study, the reaction at the anchorage unit was neglectable for both 5 mm and 10 mm activation, which is in agreement with the previous study [26]. In a clinical setting, when the anterior segment is activated for the intrusion, extrusive force, and tip back moment appear on the molar (Figure 1.) The moment leads to the molar distal tip. To avoid unwanted tooth movement in the anchorage unit, the use of skeletal anchorage is a method of choice. Insertion of a cantilever in a bracket slot of the TAD produces an undesirable couple. The cantilever’s size should match the dimensions of the slot to reduce the wire play and prevent screw failure [32]. Cantilevers can be applied to the temporary anchorage devices (TAD) for single impacted tooth traction [33], and molar uprighting [34]. Tipped molar can be directly ligated to the TAD, to neutralize vertical forces with uprighting with the cantilever hooked at the anterior segment [35]. The variation of Burstone’s three-piece intrusion arch connected to miniscrews can eliminate the side effects of conventional treatment methods and avoid unwanted movement of the anchor teeth [36,37].

### 4.2. Clinical Implications

As cantilevers deliver a statically determined force system, generated tooth movement is predictable. The segmented approach to intrusion and retraction allows simultaneous control of tooth movement in the anteroposterior and vertical planes. In case of a correction of the combined overbite and overjet, a combination of intrusion and retraction is desired [28]. TMA has desired properties for a cantilever spring: low load-deflection rate, large range of activations with less frequent reactivations needed [28]. The choice of cantilever design in a three-piece intrusion arch can help to solve different clinical issues. In patients where a combination of retraction and intrusion of incisors is desired, the use of a curved cantilever will result in an additional horizontal force component. If the protrusion is anticipated, a utility-shaped cantilever will deliver the desired combination of protrusive and intrusive forces over a long-range [1]. Another possible use is midline correction, as the cantilevers are known to produce medial and lateral forces during deactivation [26].

### 4.3. Limitations

All measurements presented in the current study were obtained in vitro. Therefore, they may not reflect clinical conditions. Classical minimum sample size or power calculation could not be performed, as there were no pre-existing variation values available from other studies, and because conventional descriptive and comparative statistics were not performed. The sample size of five copies per cantilever design was decided upon as a compromise between having on the one hand as many samples as possible and on the other hand keeping the time, it took to bend the total number of cantilevers into shape, feasible. Other external influences were minimalized by carrying out all measurements on the same day and by the same operators. In the future, the sample can be increased. The wires originated from the same package with the same reference number to avoid inequality between batches. All tests were performed in the room temperature: it will be interesting to evaluate if the results would differ in different temperatures. It may be plausible that change of the temperature may have an impact on the properties of a TMA wire. In the experimental part, the assessment of the results from the experimental part was performed qualitatively, since no measurements were obtained: no error of the method (number) could be assessed. Typodonts are an excellent tool to perform a simulation of tooth movement. However, biological aspects (periodontium, bone remodeling) are not taken into consideration and the current study should be viewed and considered as a proof of concept [38,39]. The weakness of this study is that it was performed in vitro. However, due to ethical considerations, the study could not be performed clinically on patients.

### 4.4. Recommendations for Future Research

Since the arch-formed designs produce rather low forces while the straight designs were rather high, it would be interesting to investigate how the “amount of arch-form” influence the produced force magnitude. A design in between arch-formed and straight may possibly produce optimal force magnitudes.

Furthermore, in the present study, a global coordinate system of the forces was used. It may be interesting to control to what extent an adjustment of the coordinate system according to the tangent of the wire would change the results.

## 5. Conclusions

The present study was performed with the ultimate goal to achieve new insights into the mechanics of orthodontic cantilevers, and how these can be applied in a clinical setting. As it was performed in vitro, it is subjected to multiple limitations. This laboratory study showed that considered configurations can be distinctively ranked from the deep curve, tip-back to 6 mm high utility arch according to the increasing protrusive nature of the generated sagittal forces. Simultaneous intrusion and retrusion or protrusion of the anterior segment are achieved by symmetric cantilever configurations. Apart from the vertical forces generated by the arch-formed cantilevers, an increase in the amount of activation has no proportional effect on the force levels. The most significant contributing factor to the force levels generated is whether the cantilever is shaped according to the dental arch in the occlusal view or not. As a straight cantilever requires a larger amount of activation, it will also generate higher deactivation forces.

## Figures and Tables

**Table 1:** Overview of the cantilever designs.

OCCLUSAL		Straight		Arch-Formed	
VERTICAL		5 mm	10 mm	5 mm	10 mm
**SAGITTAL**	**Tip-back**	TB_5_S	TB_10_S	TB_5_A	TB_10_A
	**Flat curve**	FC_5_S	FC_10_S	FC_5_A	FC_10_A
	**Deep curve**	DC_5_S	DC_10_S	DC_5_A	DC_10_A
	**Utility 3 mm**	UA3_5_S	UA3_10_S	UA3_5_A	UA3_10_A
	**Utility 6 mm**	UA6_5_S	UA6_10_S	UA6_5_A	UA6_10_A

**Table 2:** Overview of the generated forces in the sagittal, horizontal, and, vertical view. P: protrusive force, R: retractive force, M: medial force, L: lateral force, I: intrusive force, E: extrusive force, 0: change of force direction during deactivation.

	SAGITTAL				MEDIAL/LATERAL				VERTICAL			
	Straight		Arch-Formed		Straight		Arch-Formed		Straight		Arch-Formed	
	5 mm	10 mm	5 mm	10 mm	5 mm	10 mm	5 mm	10 mm	5 mm	10 mm	5 mm	10 mm
**Deep curve**	R	R	R	R	L	L	L	L	E	E	I	I
**Flat curve**	R	R	R	R	L	L	0	L	E	E	I	I
**Tip-back**	R	R	R	R	L	L	0	0	0	E	I	I
**Utility 3 mm**	R	R	P	R	L	L	M	0	E	E	I	I
**Utility 6 mm**	R	R	P	P	L	L	M	M	E	E	I	I

**Table 3:** Forces (cN) produced by the arch-formed cantilever configurations during deactivation.

	Deep Curve						Flat Curve						Tip-Back						Utility 3 mm						Utility 6 mm					
	5 mm			10 mm			5 mm			10 mm			5 mm			10 mm			5 mm			10 mm			5 mm			10 mm		
de- activation	Fx	Fy	Fz	Fx	Fy	Fz	Fx	Fy	Fz	Fx	Fy	Fz	Fx	Fy	Fz	Fx	Fy	Fz	Fx	Fy	Fz	Fx	Fy	Fz	Fx	Fy	Fz	Fx	Fy	Fz
**0.0 mm**	−2.9	−5.8	13.0	−9.1	−21.2	25.2	−2.3	−2.6	16.5	−3.3	−3.9	24.7	−1.7	−0.2	15.2	−2.4	0.3	25.8	2.3	8.3	14.1	−0.1	1.6	23.7	2.8	8.6	14.7	3.0	9.8	23.4
**0.5 mm**	−2.6	−4.9	11.7	−8.6	−19.8	24.2	−2.1	−2.3	15.3	−3.2	−4.0	23.4	−1.7	−0.3	13.6	−2.7	−0.6	24.2	1.5	6.3	12.3	−0.7	0.3	22.2	2.0	6.5	12.9	2.2	7.9	21.6
**1.0 mm**	−2.2	−4.1	10.3	−7.9	−18.1	23.2	−1.8	−1.9	14.1	−3.1	−3.8	22.1	−1.6	−0.4	12.1	−2.9	−1.2	22.7	0.8	4.7	10.7	−1.1	−0.8	20.8	1.4	4.9	11.3	1.6	6.4	19.9
**1.5 mm**	−1.8	−3.3	8.9	−7.2	−16.3	22.0	−1.6	−1.4	13.0	−2.8	−3.3	20.8	−1.5	−0.3	10.5	−2.9	−1.3	21.2	0.4	3.6	9.2	−1.4	−1.4	19.5	1.0	3.6	9.7	1.2	5.1	18.4
**2.0 mm**	−1.4	−2.5	7.4	−6.4	−14.2	20.8	−1.3	−1.0	11.8	−2.4	−2.5	19.4	−1.3	−0.2	9.0	−2.7	−1.0	19.8	0.1	3.0	7.8	−1.5	−1.7	18.3	0.6	2.7	8.4	0.8	4.1	17.0
**2.5 mm**	−1.1	−1.7	6.0	−5.5	−12.0	19.5	−1.0	−0.5	10.6	−1.9	−1.5	18.1	−1.1	0.0	7.6	−2.3	−0.3	18.4	0.1	2.7	6.6	−1.4	−1.6	17.2	0.4	2.2	7.1	0.5	3.5	15.8
**3.0 mm**	−0.7	−0.9	4.5	−4.6	−9.5	18.0	−0.7	0.1	9.3	−1.3	−0.2	16.7	−0.9	0.4	6.1	−1.8	0.8	17.1	0.3	2.9	5.4	−1.2	−1.1	16.2	0.3	2.0	6.0	0.4	3.1	14.7

**Table 4:** Forces (cN) produced by the straight cantilever configurations during deactivation.

	Deep Curve						Flat Curve						Tip-Back						Utility 3 mm						Utility 6 mm					
	5 mm			10 mm			5 mm			10 mm			5 mm			10 mm			5 mm			10 mm			5 mm			10 mm		
de- activation	Fx	Fy	Fz	Fx	Fy	Fz	Fx	Fy	Fz	Fx	Fy	Fz	Fx	Fy	Fz	Fx	Fy	Fz	Fx	Fy	Fz	Fx	Fy	Fz	Fx	Fy	Fz	Fx	Fy	Fz
**0.0 mm**	−299.9	−340.9	−58.1	−255.8	−299.2	−67.2	−276.8	−311.6	−49.8	−258.3	−293.0	−48.2	−252.6	−278.8	−20.9	−203.4	−229.5	−32.8	−113.0	−98.6	−21.0	−116.6	−104.5	−11.5	−57.2	−48.3	−11.8	−54.2	−45.5	−1.2
**0.5 mm**	−304.5	−346.0	−52.4	−257.5	−301.7	−62.7	−281.8	−317.3	−44.4	−261.1	−295.2	−43.8	−259.7	−286.8	−16.8	−207.0	−233.0	−29.7	−119.2	−104.2	−21.0	−120.4	−108.0	−10.8	−61.0	−52.0	−12.8	−57.0	−48.3	−1.9
**1.0 mm**	−308.7	−349.9	−46.4	−259.3	−304.2	−57.7	−286.2	−321.8	−38.8	−264.0	−297.4	−38.8	−265.9	−293.1	−12.3	−210.5	−236.4	−26.2	−124.9	−109.0	−20.4	−124.0	−111.1	−9.8	−64.6	−55.3	−13.5	−59.7	−50.8	−2.3
**1.5 mm**	−312.5	−352.7	−40.1	−261.1	−306.6	−52.3	−290.0	−325.0	−33.0	−266.9	−299.8	−33.4	−271.2	−297.8	−7.5	−213.8	−239.5	−22.3	−130.1	−113.1	−19.5	−127.5	−114.0	−8.5	−67.9	−58.2	−13.9	−62.2	−53.2	−2.5
**2.0 mm**	−315.8	−354.2	−33.5	−262.9	−309.0	−46.5	−293.2	−326.9	−27.0	−269.8	−302.3	−27.6	−275.7	−300.8	−2.4	−217.0	−242.3	−18.0	−134.7	−116.3	−18.1	−130.7	−116.5	−6.9	−71.0	−60.7	−13.9	−64.6	−55.3	−2.5
**2.5 mm**	−318.7	−354.6	−26.7	−264.7	−311.5	−40.3	−295.9	−327.6	−20.7	−272.9	−304.8	−21.3	−279.3	−302.3	3.1	−220.0	−245.0	−13.4	−138.8	−118.8	−16.2	−133.8	−118.7	−5.1	−73.8	−62.9	−13.7	−66.8	−57.1	−2.4
**3.0 mm**	−321.1	−353.8	−19.5	−266.5	−313.8	−33.7	−298.0	−327.0	−14.3	−276.0	−307.5	−14.5	−282.0	−302.1	8.9	−222.8	−247.4	−8.4	−142.4	−120.5	−13.9	−136.6	−120.5	−3.0	−76.4	−64.7	−13.2	−68.9	−58.8	−2.0

## Data Availability

Not applicable.
